# Dermoscopy of Umbilical Lesions—A Systematic Review

**DOI:** 10.3390/jcm13061790

**Published:** 2024-03-20

**Authors:** Jakub Żółkiewicz, Martyna Sławińska, Urszula Maińska, Roman J. Nowicki, Michał Sobjanek, Luc Thomas

**Affiliations:** 1 Department of Dermatology, Venereology and Allergology, Faculty of Medicine, Medical University of Gdańsk, 80-214 Gdańsk, Poland; dr.jakubzolkiewicz@gmail.com (J.Ż.); ulakobus@gumed.edu.pl (U.M.); rnowicki@gumed.edu.pl (R.J.N.); michal.sobjanek@gumed.edu.pl (M.S.); 2 Department of Dermatology, Centre Hospitalier Lyon Sud, 69495 Lyon, France; luc.thomas@chu-lyon.fr; 3 Lyon 1 University, 69000 Lyon, France; 4 Lyons Cancer Research Center UMR INSERM U1052–CNRS5286–UCBL1, 69008 Lyon, France

**Keywords:** dermoscopy, dermatoscopy, melanoma, nevus, Sister Mary Joseph nodule, endometriosis, umbilicus, navel

## Abstract

**Background**: The umbilicus is a fibrous remnant located in the centre of the abdomen. Various entities may be encountered in this special anatomical location; however, little is known about their dermoscopic presentation. The aim of this study was to provide a comprehensive summary of existing evidence on dermoscopic features of umbilical lesions. **Methods**: Studies assessing dermoscopic images of umbilical lesions were included in this study. No age, ethnicity or skin phototype restrictions were applied. Papers assessing lesions outside of the umbilical area, lacking dermoscopic images and/or dermoscopic description and not related to the topic were excluded. Embase, Medline and Cochrane Library were searched from inception to the end of May 2023. The Joanna Briggs Institute critical appraisal tools were used to evaluate the risk of bias of the selected studies. The quality and the level of evidence of included studies were assessed according to the Oxford 2011 Levels of Evidence. Thirty-four studies reporting a total of 39 lesions met the inclusion criteria and were included in qualitative analysis. **Results**: A qualitative synthesis of the following entities was performed: melanoma, nevi, basal cell carcinoma, fibroepithelioma of Pinkus, Sister Mary Joseph nodule, mycosis fungoides, dermatofibroma, endometriosis, epidermal cyst, granuloma, intravascular papillary endothelial hyperplasia, lichen planus, omphalolith, seborrheic keratosis, and syringoma. **Conclusions**: Dermoscopy is a non-invasive technique that may be useful in the differential diagnosis of umbilical lesions. The main limitations of this study were lack of a high level of evidence in the studies and the lack of uniformity in applied dermoscopic terminology between included studies.

## 1. Introduction

Dermoscopy is a non-invasive magnification technique that allows for more detailed skin examination compared to the naked eye. Although dermoscopic images are not always indicative of underlying lesions, specific dermoscopic structures may facilitate their differentiation and recognition. One of the important variables that may affect dermoscopic presentation seems to be anatomical location. Among such special locations that require a peculiar dermoscopic approach are the facial and acral areas, as well as the nail apparatus region [1].

Embryologically, the umbilicus serves as an opening for blood vessels supplying the foetus with oxygen and essential nutrients, and it is involved in the development of the gastrointestinal and urinary tracts. After birth, the umbilicus undergoes involution, forming a fibrous remnant on the anterior abdominal wall. The umbilicus is the only physiologically occurring scar on the human body; however, due to existing embryological remnants and the presence of biologically active tissues, it is prone to various clinical entities [2]. In this special anatomical location, inflammatory disorders, as well as benign and malignant tumors, may be encountered [3].

Little is known about clinical and dermoscopic aspects of dermatological disorders affecting this special location. Moreover, the anatomical heterogeneity of umbilici exhibiting various shapes, curvatures or protrusions impedes the technical aspect and interpretation of dermoscopic pictures.

To date, dermoscopic features of umbilical lesions have not been systematically evaluated. The aim of this study was to analyse and summarize literature data on the dermoscopic presentation of different entities affecting the umbilical area.

## 2. Materials and Methods

This systematic review was performed in accordance with the PRISMA (Preferred Reporting Items for Systematic Reviews and Meta Analyses) guidelines. Embase, Medline and Cochrane Library were searched from inception to the end of May 2023. The search strategy was written for the Medline database and was adapted to the respective database syntax. The following search query was used: “(umbilic* OR belly button OR navel) AND (epilum* OR dermo* OR dermato*)”. No filters were used.

Original studies and case reports assessing dermoscopic images of umbilical lesions were included in this study. No age, ethnicity or skin phototype restrictions were applied. Papers assessing lesions outside of the umbilical area, lacking dermoscopic images and/or dermoscopic description and not related to the topic were excluded. We included all types of study design, as we anticipated that, due to the low incidence of umbilical lesions, there would be few suitable randomized controlled trials or comparative studies.

**Figure 1:**
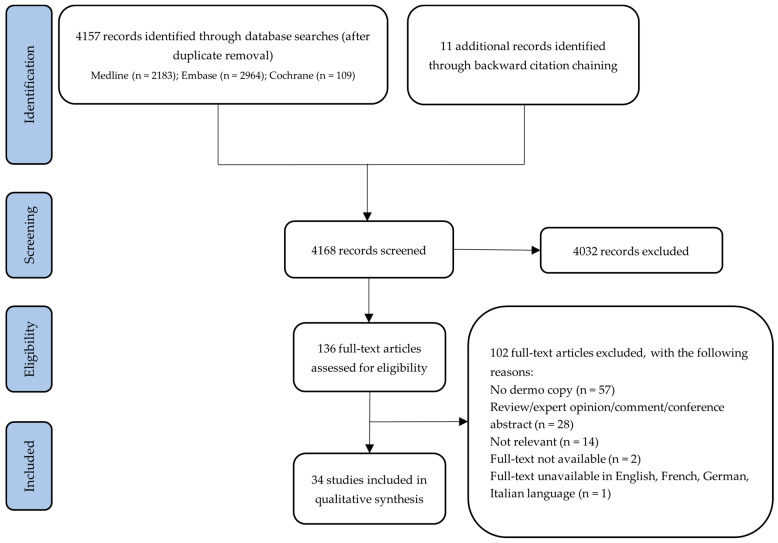
Flow chart of the literature search and study selection process.

After duplicate removal, all data were extracted independently by two investigators (J.Ż. and M.S.), and any disagreements were resolved by a third reviewer (M.So.). Backward citation chaining of included studies was performed to find further relevant publications. Review articles, personal opinions/editorials, conference abstracts, and articles in languages other than English, French, German, and Italian were excluded. A flow chart reporting the study selection process is presented below (Figure 1).

Apart from dermoscopic features, the number of cases, corresponding histopathology, type of dermatoscope used, magnification, polarization mode, and application of immersion were also retrieved. The Joanna Briggs Institute (JBI) critical appraisal tools [4] were used by two investigators (J.Ż. and M.S.), who independently evaluated the risk of bias of the selected studies. The JBI checklists utilized for the purpose of this review are described in Supplementary Table S1. The risk of bias of each study was considered as “low” if it met >70% of the analysed criteria, “moderate” when >50% of criteria were met, and “high” for the remaining cases. The quality and the level of evidence of included studies were assessed independently by two investigators (J.Ż. and M.S.) according to the Oxford 2011 Levels of Evidence [5].

## 3. Results

The initial database search identified 4157 unique records. Eleven additional studies were identified through backward citation chaining. After screening on the basis of title and abstract, 136 papers were selected for full-text appraisal. A total of 34 studies (33 case reports and 1 cross-sectional study) reporting a total of 39 lesions met the inclusion criteria and were included in the qualitative analysis. A summary of the screening process is presented in the above-mentioned flow chart. The following entities were identified and evaluated in this study: melanoma, nevi, basal cell carcinoma, fibroepithelioma of Pinkus, Sister Mary Joseph nodule, mycosis fungoides, dermatofibroma, endometriosis, epidermal cyst, granuloma, intravascular papillary endothelial hyperplasia, lichen planus, omphalolith, seborrheic keratosis, and syringoma.

The type of dermatoscope was specified in 9/34 papers (26.5%), magnification was mentioned in 10/34 studies (29.4%) and polarization was indicated in 9/34 articles (26.5%). One of the included studies mentioned the type of immersion used. In 34 out of 39 cases (87.2%) the diagnosis was confirmed by histopathological examination.

One cross-sectional study [6] did not provide a detailed frequency of the dermoscopic features of each of included type of lesion. Therefore, we analysed the dermoscopic patterns of single cases and individual cases of multiple lesions that had their dermoscopic image explicitly described in the figure caption.

The risk-of-bias assessment of included studies is presented in Supplementary Table S2. In all papers except one [7], the risk of bias was assessed as low (33/34; 61.8%). The paper by Gracia-Darder [7] was published as a photo submission (“Visual Dermatology”), and despite the lack of detailed patient data, the article was included in the qualitative synthesis as it meticulously described dermoscopic features of the underlying condition.

Table 1 summarizes the dermoscopic features of the included studies. The remaining details of the analysed studies are provided in Supplementary Table S3.

### 3.1. Melanocytic Lesions

#### 3.1.1. Melanoma

**Figure 2:**
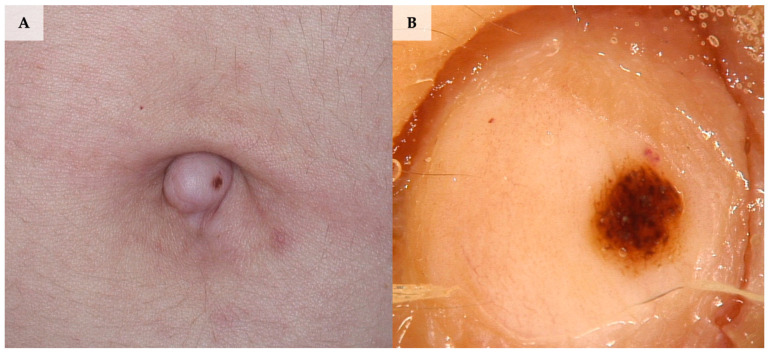
Umbilical melanoma in situ diagnosed during videodermoscopic follow-up as a new-onset lesion. (**A**) Clinical presentation. (**B**) Dermoscopy shows a globular, relatively symmetric pattern with a discrete pigmented network at the periphery (FotoFinder, Medicam 800 HD, FotoFinder Systems GmbH, Bad Birnbach, Germany; ×20 magnification, immersion gel).

Although there are currently several case reports concerning dermoscopy of umbilical melanoma, the only one fitting inclusion criteria was published by Campos-Muñoz et al. [8]. The lesion was primarily misdiagnosed with omphalitis due to umbilical discharge. Dermoscopy showed an atypical pigment network, homogenous blue-grey background in the centre, and brownish pigmentation with an atypical pigment network, and some dark-brown globules at the periphery (Figure 2).

#### 3.1.2. Nevi

**Figure 3:**
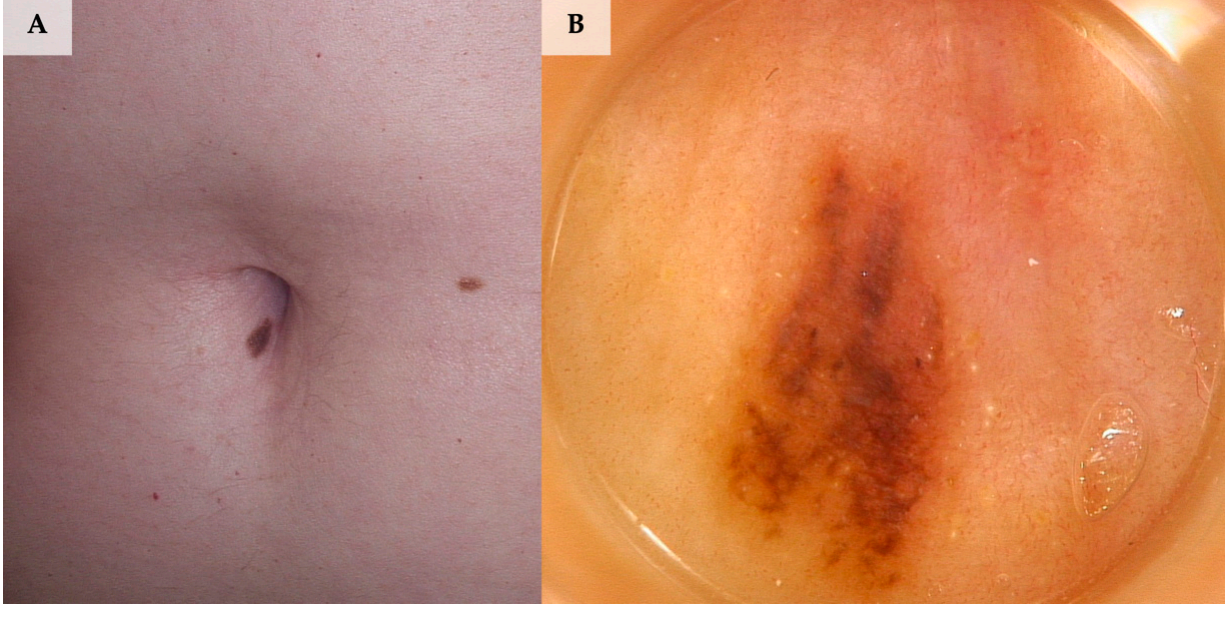
Umbilical compound nevus. (**A**) Clinical presentation. (**B**) Dermoscopy shows multicomponent pattern with structureless areas, angulated lines and yellow globules at the periphery (FotoFinder, Medicam 800 HD, FotoFinder Systems GmbH, Bad Birnbach, Germany; ×20 magnification, immersion gel).

**Figure 4:**
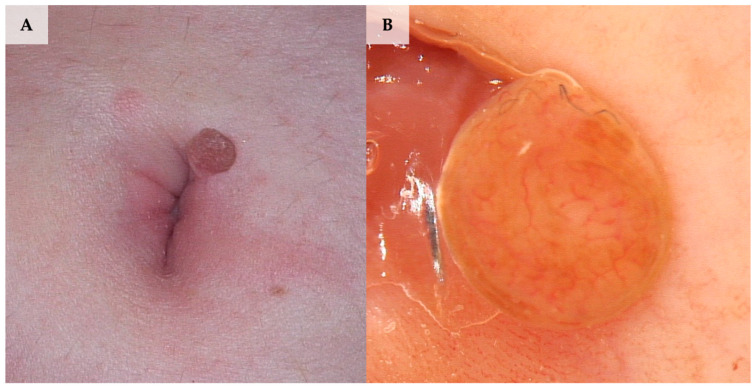
Umbilical dermal nevus. (**A**) Clinical presentation. (**B**) Dermoscopy shows branching, pink, poorly demarcated vessels over light-brownish background (FotoFinder, Medicam 800 HD, FotoFinder Systems GmbH, Bad Birnbach, Germany; ×20 magnification, immersion gel).

**Figure 5:**
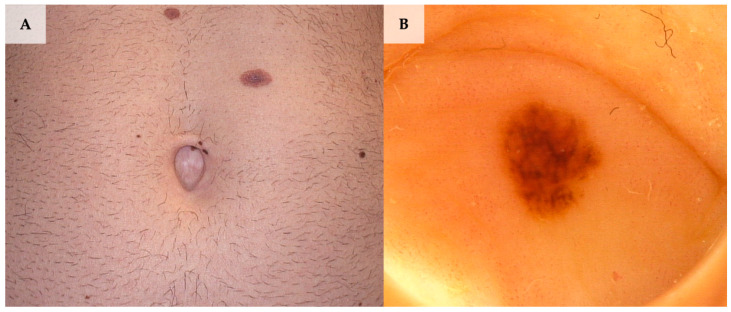
Compound nevus (with a predominant junctional component) of the umbilicus in a patient with atypical mole syndrome. (**A**) Clinical presentation. (**B**) Dermoscopy shows small, hyperpigmented patches and angulated lines over a brown, structureless background (FotoFinder, Medicam 800 HD, FotoFinder Systems GmbH, Bad Birnbach, Germany; ×20 magnification, immersion gel).

The systematic review revealed 2 case reports of melanocytic nevi located in the umbilicus, namely congenital melanocytic nevus and intradermal nevus. The umbilical congenital nevus described by Drakensjö et al. [9] exhibited irregularly distributed black and brown dots, which faded over follow-up period until a minor residual brown macula with a greyish shadow remained. The intradermal nevus reported by Bandeira et al. [10] was the only case of umbilical nevus showing a vascular pattern (consisting of comma-shaped and irregular vessels) with no associated pigmented structures. Apart from the aforementioned features, the lesion exhibited a polymorphous dermoscopic pattern consisting of a cerebriform area and a light-pink background accompanied by a serous and hematic crust (Figure 3, Figure 4 and Figure 5).

### 3.2. Non-Melanocytic Malignant Lesions

#### 3.2.1. Basal Cell Carcinoma

Despite basal cell carcinoma (BCC) being the most frequent skin cancer, an umbilical location of BCC is extremely rare. To date, fewer than 20 cases have been reported, with only four, herein reviewed, including dermoscopic presentation. Ramirez et al. [11] described semi-translucency, small superficial ulceration, and a polymorphous vascular pattern (hairpin and arborizing vessels as well as fine elongated telangiectasias). In another umbilical case of BCC reported by Takada et al. [12], dermoscopy showed large blue-grey ovoid nests. Two additional cases were provided by Yukiho et al. [13], both of which manifested with large blue-grey ovoid nests in dermoscopy. Moreover, blue-grey globules and arborizing vessels were also identified in this small series of two cases. The dermoscopic features presented in the above-mentioned papers correspond to those found in BCCs located in other anatomical regions.

#### 3.2.2. Fibroepithelioma of Pinkus

Fibroepithelioma of Pinkus (FEP) is an uncommon skin neoplasm that is classified as a variant of BCC. However, due to the presence of Merkel cells within the tumour, which are typically absent in BCC, some authors consider it a trichoblastoma [40]. The literature search identified two FEP lesions in one patient described by Inskip et al. [14]. Both tumours were histopathologically confirmed as two distinct lesions, one of which was pigmented, while the other was a nonpigmented variant of FEP. Pigmented lesion revealed small ovoid grey structures, fine brown dots, arborizing vessels, and a single erosion. Nonpigmented lesion showed multiple small erosions along with fine polymorphic peripheral vessels on a pink/white background. These features are generally within the dermoscopic spectrum of BCC.

#### 3.2.3. Sister Mary Joseph Nodule

Sister Mary Joseph nodule (SMJN) is an eponymous term for umbilical skin metastases. The umbilicus is prone to cutaneous metastases, since it is devoid of a muscle layer [18]. However, other possible mechanisms of tumour spread have been proposed, including via arterial, venous and lymphatic systems, with which the umbilicus is inseparably connected. Presumably, the exact route of malignant cell dissemination also depends on the histologic nature and location of the primary neoplasm.

The systematic review revealed 7 cases of SMJN in seven studies [6,7,15,16,17,18,19]. The most prevalent primary location of tumours was the stomach–2 out of 7 cases (28.6%) originated from gastric adenocarcinoma. The other cases were metastases from colon cancer, pancreatic cancer and oesophageal squamous cell carcinoma (1/7; 14.3%). In two cases, the primary site was not specified (2/7; 28.6%). In all cases, the authors described the presence of polymorphous vessels (7/7; 100%) with serpentine vessels (3/7; 42.9%) and curved vessels (3/7; 42.9%) encountered most frequently. Milky red areas as well as white structureless areas were described in half of the reported cases (4/7; 57.1%). Furthermore, Dong et al. [16] reported peculiar SMJN presentation described as a ‘frogspawn appearance’.

#### 3.2.4. Mycosis Fungoides

Mycosis fungoides (MF) is a rare lymphoproliferative disorder that primarily affects the skin. One dermoscopic case of umbilical MF has been identified in the literature [20]. The patient had a history of breast cancer, therefore, the umbilical tumour was initially considered a metastasis (SMJN). Although dermoscopy revealed the presence of comedo-like openings over a black structureless area (suggestive of seborrheic keratosis), histopathological examination, together with immunostaining, confirmed the diagnosis of plaque MF.

### 3.3. Non-Melanocytic Benign Lesions

#### 3.3.1. Dermatofibroma

Dermatofibroma (DF), also known as fibrous histiocytoma, is a benign, dermal-based neoplasm of unknown aetiology [41]. Only one dermoscopic case of umbilical DF has been published, which revealed a pigment network and a white structureless area [6]. This is within the known dermoscopic spectrum of DF [42].

#### 3.3.2. Endometriosis

The systematic review revealed 10 papers reporting 10 cases of umbilical endometriosis [15,21,22,23,24,25,26,27,28,29]. In such cases, a clinical history indicating lesion evolution during the menstrual cycle may serve as an important diagnostic clue, however, this clinical sign was revealed in only 2 out of 10 included cases (20%) [15,22]. Parallel to changing clinical presentation during the menstrual cycle, alterations in dermoscopic appearance may be observed, which was shown in the case report by Costa et al. [22].

The three most prevalent dermoscopic findings were red globules, polyploid nodules and homogeneous reddish pigmentation, usually fading toward the periphery of the lesion—all features were present in 3 out of 10 cases (30%). In two cases (2/10; 20%), the authors described specific dermoscopic presentation of ‘red atolls’, which were defined as small red globular structures within homogeneous reddish pigmentation. Dermoscopically, brown structureless areas and milky-red areas were associated with endometriosis in 2/10 of lesions (20%). Furthermore, Bonné et al. [21] showed a dermoscopic manifestation of umbilical endometriosis characterized by polyploid nodules with a drainage hole on the top of each nodule. Drainage holes were encircled by purple-blue structureless area and one of the holes was filled with amorphous red material.

Most of reported cases did not provide information regarding the phase of the menstrual cycle at the time when dermoscopy was performed. The corresponding menstrual phase was mentioned in 3 out of 10 papers (30%)—in two cases it was established based on the histopathological features [24,25] and in one case based on anamnesis [22]. The case report by Costa et al. [22] was the only to describe different dermoscopic features of umbilical endometriosis depending on the phase of the menstrual cycle, revealing volume increase of polyploid projections along with increase in the number and size of the dark-brown spots in the luteal phase when compared to the same lesion in the follicular phase. The luteal phase was also distinguished by the presence of areas of dark material, which were the remnants of active bleeding, which, in turn, was noted in the follicular phase.

#### 3.3.3. Epidermal Cyst

An epidermal cyst involving the umbilicus was described in one cross-sectional study by Ha et al. [6]. In this case, dermoscopy allowed for the identification of a “pore sign”, which corresponds with follicular ostium and also serves as a diagnostic clue in other anatomical locations [43].

#### 3.3.4. Epidermal Nevus

Two dermoscopic cases have been identified in the umbilical area. Siebel et al. [30] described the presence of a pigment network, centrally located yellow-orange structureless areas along with a scar and a verrucous appearance at the periphery. The descriptive dermoscopy of the umbilical epidermal nevus provided by Ha et al. [6] was scanty and embraced only the presence of diffuse pigmentation, with no other pattern shown.

#### 3.3.5. Granuloma

The systematic review revealed two cases of umbilical granuloma. Both of them (2/2; 100%) presented with a polymorphic vascular pattern and a milky-white background, corresponding with the presence of granulation tissue and the proliferation of vascular endothelial cells and fibroblasts [31,32].

#### 3.3.6. Intravascular Papillary Endothelial Hyperplasia

Intravascular Papillary Endothelial Hyperplasia (IPEH), also known as Masson’s tumour, is a benign lesion of vascular origin. It has been suggested that IPEH may not actually be a tumour but a reactive proliferation of the vascular endothelial cells triggered by a traumatic vascular stasis or thrombus. Resuello et al. [33] were the first and only to illustrate a dermoscopic picture of umbilical IPEH. The authors noted the presence of a patchy pseudo-reticular network with thick brown lines surrounding a structureless area. Furthermore, a milky-red structureless area, white veil, and multiple shiny white lines and strands were observed.

#### 3.3.7. Lichen Planus

**Figure 6:**
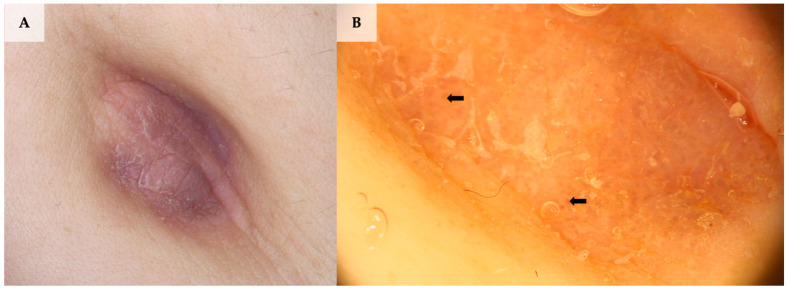
Lichen planus involving the umbilical area. (**A**) Clinical presentation. (**B**) Dermoscopy shows brown structureless pattern, white lines (Wickham striae; black arrows), coiled vessels and white scale (FotoFinder, Medicam 800 HD, FotoFinder Systems GmbH, Bad Birnbach, Germany; ×20 magnification, immersion gel).

Two dermoscopic case reports of lichen planus (LP) with umbilical localization have been described [34,35]. In both patients, white lines over a pink/violaceous background were identified. In addition, in the case of lichen planus pigmentosus inversus, dermoscopy showed grey dots, as well as a grey-black structureless area (Figure 6).

#### 3.3.8. Omphalolith

Two cases of omphalolith encompassing dermoscopy have been reported in the literature. Gallouj et al. [36] delineated the presence of black-brown structureless areas along with a dry crusted appearance. The authors highlighted the fact that neither a pigment network, dots, globules nor a vascular pattern were present, what supported the differential diagnosis. In another dermoscopic case of omphalolith provided by Jouini et al. [37] the presence of dry crusted pigmented lamellar keratotic material was reported.

#### 3.3.9. Seborrheic Keratosis

**Figure 7:**
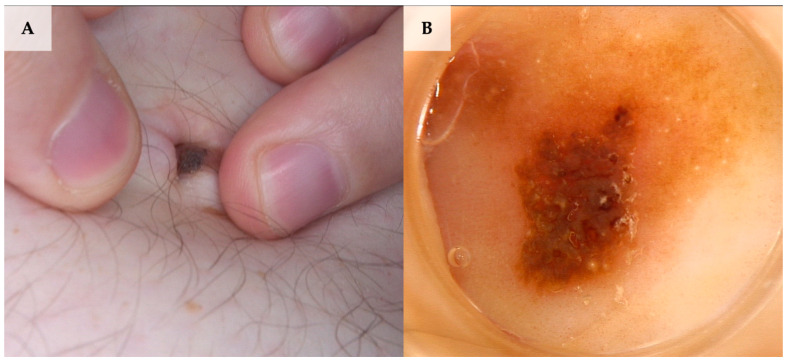
A collision of dermal nevus and reticular seborrheic keratosis. (**A**) Clinical presentation. (**B**) Dermoscopy shows structures corresponding to both seborrheic keratosis and dermal nevus, namely dark-brown structureless areas, thick curved lines (fissures and crypts), and white/yellow disseminated dots/clods (milia-like cysts) in seborrheic keratosis and a light-brown pigmented network and structureless areas along with dotted vessels and white/yellow disseminated dots/clods (milia-like cysts) in dermal nevus (FotoFinder, Medicam 800 HD, FotoFinder Systems GmbH, Bad Birnbach, Germany; ×20 magnification, immersion gel).

When it comes to seborrheic keratosis, the systematic review revealed a single case that met the inclusion criteria. The dermoscopic image presented by Hamich et al. [38] was not specific and comprised an exophytic keratotic projection pattern with dotted vessels, brown and yellowish structureless areas, and aggregated blue-grey globular-like structures (Figure 7).

#### 3.3.10. Syringoma

Syringomas are benign tumours derived from sweat glands and usually located on the eyelids and in the malar region. Although syringomas have already been described in various anatomical locations, umbilical manifestation has been reported in only one case by Nam et al. [39]. Dermoscopic findings outlined by the authors included multiple pink to whitish areas surrounded by a finely pigmented network. Similar structures were also encountered in other anatomical locations [44].

## 4. Discussion

In summary, this systematic review provides a comprehensive overview of the current knowledge on dermoscopic features of various umbilical lesions. One of the main limitations of this systematic review was low number of reported cases and the lack of studies with a high level of evidence, as almost all extracted data comes from case reports.

Interestingly, most reports concerned tumours, whilst only two papers devoted to inflammatory dermatosis, namely LP, were identified. Dermoscopic patterns of some entities (e.g., DF, epidermal cyst, LP, syringoma) seem to correspond to those observed in other anatomical locations. Similarly, differentiation of some clinical conditions in this special anatomical area is not possible due to the overlap of dermoscopic features (e.g., FEP, metastatic tumours, MF, granuloma, IPEH presenting with polymorphic vessels and white-red/pink structureless areas).

More evidence was extracted from reports concerning cutaneous endometriosis and SMJN. Despite some differences in the dermoscopic presentation of both conditions (polymorphic vessels in SMJN and a combination of bluish and brown structureless areas in endometriosis), the final diagnosis cannot be established based on dermoscopy. In such cases, periodical alternations in clinical and dermoscopic presentation may be supportive [22].

Another concern that emerged while conducting this study was lack of uniformity in applied dermoscopic terminology between included studies, therefore, corresponding terminology proposed by the IDS consensus has been added in the Supplementary Table S3 [45]. Additionally, some specific clues not described in the consensus were also involved.

## 5. Conclusions

Dermoscopy should be considered as an auxiliary tool facilitating differential diagnosis of umbilical lesions, complementary to clinical evaluation. Until now, neither prospective nor retrospective original study concerning the subject matter of this systematic review has been published. More data should be scrutinized in order to establish more decisive conclusions regarding specific dermoscopic patterns of umbilical skin lesions.

## Figures and Tables

**Table 1:** Summary of reported dermoscopic features of included studies.

Diagnosis	First Author, Journal, Year	Dermoscopic Features		Number of Cases
**Melanocytic lesions**				
**Melanoma**	Campos-Muñoz et al., Clin Exp Dermatol., 2007 [8]	Homogeneous blue-grey background with a superimposed atypical pigment network at the centre.		1
		Brownish pigmentation with an atypical pigment network.		
		Dark-brown globules at the periphery.		
**Congenital melanocytic naevus**	Drakensjö et al., Acta Derm Venereol., 2012 [9]	Irregular pigment distribution with confluence of black and brown dots.		1
**Intradermal nevus**	Bandeira et al., An Bras Dermatol., 2018 [10]	Comma-shaped and irregular vessels on a light-pink background. Surrounding serous and hematic crust.		1
**Non-melanocytic malignant lesions**				
**Basal cell carcinoma**	Ramirez et al., Dermatol. Online J., 2011 [11]	Semi-translucent appearance.		1
		Small superficial ulceration.		
		Polymorphous in focus vessels: hairpin vessels and prominent arborizing vessels.		
		Fine elongated telangiectasias.		
	Takada et al., An Bras Dermatol., 2021 [12]	Large blue-grey ovoid nests.		1
	Kurosaki et al., Medicine, 2023 [13]	Case 1.	Blue-grey globules.	2
			Large blue-grey ovoid nests.	
		Case 2.	Large blue-grey ovoid nests.	
			Arborizing vessels.	
**Fibroepithelioma of Pinkus**	Inskip et al., Dermatol Pract Concept., 2016 [14]	Lesion 1. (nonpigmented)	Multiple small erosions	1
			Fine polymorphic peripheral vessels on a pink/white background.	
		Lesion 2. (pigmented)	Small grey ovoid structures.	
			Fine brown dots.	
			Arborizing vessels.	
			Single erosion.	
**Sister Mary Joseph nodule**	Buljan et al., Australas J Dermatol., 2019 [15]	Diffuse glomerular and dotted vessels.		1
		Milky-red structureless areas.		
		White lines (as a result of the papillomatous surface of the lesion).		
	Dong et al., Australas J Dermatol., 2016 [16]	Aggregated frogspawn appearance with grey, yellow-grey, and pink-grey colouration, sometimes with a vessel in each ‘egg’ in the centre; polymorphous vessels inside ‘eggs’ (irregular linear, arborizing, glomerular vessels).		1
		Peripherally structureless pink area.		
	Garrido Colmenero et al., Actas Dermosifiliogr., 2015 [17]	White shiny structures.		1
		Milky-red areas.		
		Atypical polymorphous vessels (serpentine, curved vessels).		
	Ge et al., Dermatol Sin., 2016 [18]	Polymorphous vessels (serpentine, dotted, comma-shaped vessels).		1
		White veil.		
	Gracia-Darder et al., J Cutan Med Surg., 2022 [7]	Milky-red structureless areas.		1
		Yellow crust.		
		White lines.		
		Atypical polymorphic vascular pattern.		
	Ha et al., Clin Med Insights Oncol., 2021 [6]	Polymorphous vessels. White, milky-red structureless area.		1
	Mun et al., J Am Acad Dermatol., 2013 [19]	Polymorphous vessels (linear serpentine and linear curved vessels).		1
		White and milky-red structureless area.		
**Mycosis fungoides**	Belcadi et al., JAAD Case Rep., 2022 [20]	Comedo-like openings. Black structureless area.		1
**Non-melanocytic benign lesions**				
**Dermatofibroma**	Ha et al., Clin Med Insights Oncol., 2021 [6]	Pigment network. White structureless area.		1
	Bonné et al., J Eur Acad Dermatol Venereol., 2020 [21]	Three polypoid nodules; drainage hole on the top of each nodule; one drainage hole filled with amorphous red material; purplish-blue homogenous and poorly delimited area around the drainage holes.		1
		Flesh-coloured, light- to dark-brown structureless areas.		
	Buljan et al., Australas J Dermatol., 2018 [15]	Diffuse and homogeneously distributed dotted vessels.		1
		Milky-red structureless area with a brownish hue.		
	Costa et al., Int J Dermatol., 2014 [22]	Follicular phase	Polypoid projections of erythematous violaceous colour; active bleeding. Areas with dark-brown spots.	1
		Luteal phase	Volume increase in the polypoid projections; increase in the number and size of the dark-brown spots; areas with dark material.	
	Amaral Couto et al., Int J Clin Case Rep Rev., 2020 [23]	Polypoid projections in white colour.		1
		Greyish area around polyploid projections.		
		Central black dots.		
		Peripheral mild erythema.		
	De Giorgi et al., Clin Exp Dermatol., 2003 [24]	Greyish area around polyploid projections.		1
	Jaime et al., An Bras Dermatol., 2013 [25]	Central black dots.		1
		Peripheral mild erythema.		
		Regular pigmented skin network in the centre.		
	Levakov et al., Acta Dermatovenerol Croat., 2020 [26]	Small red globular structures (“red atolls”) within regularly distributed homogeneous reddish pigmentation fading at the periphery.		1
	Sandoval et al., Australas J Dermatol., 2021 [27]	Pink homogeneous lesion with a focal bluish blotch/clod.		1
	Vega-Castillo et al., Dermatol Pract Concept., 2022 [28]	Central white reticular pattern on a violet background.		1
	Wobser et al., Geburtshilfe und Frauenheilkunde, 2009 [29]	Red globules.		1
		Telangiectasias.		
		Diffuse, homogenous milky-red area.		
**Epidermal cyst**	Ha et al., Clin Med Insights Oncol., 2021 [6]	The ‘pore’ sign.		1
**Epidermal nevus**	Ha et al., Clin Med Insights Oncol., 2021 [6]	Diffuse pigmentation.		1
	Siebel et al., Surg Cosmet Dermatol., 2014 [30]	Yellow-orange colour areas in the centre.		1
		Peripheral verrucous lesions (on the wall of the umbilical scar).		
		Fine regular pigmented network in the margins of the lesion.		
		Debris.		
**Granuloma**	Ancer-Arellano et al., Pediatr Dermatol., 2019 [31]	Linear irregular vessels.		1
		Arborizing vessels.		
		Structureless areas over a milky-red background.		
	Jassi et al., Indian Dermatol Online J., 2020 [32]	Fine linear vessels.		1
		Dotted vessels.		
		Tortuous vessels.		
		Milky-white background.		
**Intravascular Papillary Endothelial Hyperplasia**	Resuello et al., Diagn Pathol., 2022 (under review) [33]	Patchy pseudo-reticular network with thick brown lines surrounding a structureless area.		1
		Milky-red structureless area.		
		White veil.		
		Multiple shiny white lines and strands.		
**Lichen planus**	Martos-Cabrera et al., An Bras Dermatol., 2023 [34]	White streaks on a violaceus background.		1
	Kołcz et al., Forum Dermatologicum, 2023 [35]	Irregular pinkish structureless areas intermingled with whitish lines.		1
		Grey dots.		
		Grey-black structureless area.		
		Peripheral white lines resembling Wickham striae.		
**Omphalolith**	Gallouj et al., Dermatol Online J., 2014 [36]	Dry, crusted appearance.		1
		Black-brown structureless areas.		
	Jouini et al., Clin Case Rep., 2022 [37]	Dry, crusted pigmented lamellar keratotic material.		1
**Seborrheic Keratosis**	Hamich et al., Clin Med Img Lib., 2022 [38]	Exophytic keratotic projection pattern.		1
		Dotted vessels.		
		Brown and yellowish structureless areas.		
		Aggregated blue-grey globular-like structures.		
**Syringoma**	Nam et al., Ann Dermatol., 2020 [39]	Multiple pink to whitish areas surrounded by a finely pigmented network.		1

## Data Availability

No new data were created.

## Supplementary Materials

The following supporting information can be downloaded at: https://www.mdpi.com/article/10.3390/jcm13061790/s1, Table S1: Joanna Briggs Institute Appraisal tool questions; Table S2: Risk of bias assessment; Table S3: Overview of the characteristics of included studies. References [6,7,8,9,10,11,12,13,14,15,16,17,18,19,20,21,22,23,24,25,26,27,28,29,30,31,32,33,34,35,36,37,38,39] are cited in the Supplementary Materials.
