# Supplementary material for: Dermoscopy of Umbilical Lesions—A Systematic Review
Source: J Clin Med. 2024 Mar 20;13(6):1790. doi: 10.3390/jcm13061790 (PMC10970748; doi:10.3390/jcm13061790)
Supplement: Supplementary file 1 [file jcm-13-01790-s001.zip › Supplementary_Table_S1.pdf]

Supplementary Table 1. Joanna Briggs Institute Appraisal tool questions.

Type of assessed study	Q1	Q2	Q3	Q4	Q5	Q6	Q7	Q8
**Analytical cross-sectional study**	Were the criteria for inclusion in the sample clearly defined?	Were the study subjects and the setting described in detail?	Was the exposure measured in a valid and reliable way?	Were objective, standard criteria used for measurement of the condition?	Were confounding factors identified?	Were strategies to deal with confounding factors stated?	Were the outcomes measured in a valid and reliable way?	Was appropriate statistical analysis used?
**Case report**	Were patient's demographic characteristics clearly described?	Was the patient's history clearly described and presented as a timeline?	Was the current clinical condition of the patient on presentation clearly described?	Were diagnostic tests or assessment methods and the results clearly described?	Was the intervention (s) or treatment procedure(s) clearly described?	Was the post-intervention clinical condition clearly described?	Were adverse events (harms) or unanticipated events identified and described?	Does the case report provide takeaway lessons?

Q – question
