# Supplementary material for: Dermoscopy of Umbilical Lesions—A Systematic Review
Source: J Clin Med. 2024 Mar 20;13(6):1790. doi: 10.3390/jcm13061790 (PMC10970748; doi:10.3390/jcm13061790)
Supplement: Supplementary file 1 [file jcm-13-01790-s001.zip › Supplementary_Table_S2 final.pdf]

Supplementary Table 2. Risk of bias assessment.

First author, year	JBI tool used	Q1	Q2	Q3	Q4	Q5	Q6	Q7	Q8	Results (% of yes answers)	Risk of bias
Amaral Couto, 2020 [23]	CR	+	+	+	-	+	+	U	+	85.7%	low
Ancer-Arellano, 2019 [31]	CR	+	+	+	+	-	+	U	+	85.7%	low
Bandeira, 2018 [10]	CR	+	+	+	-	+	+	U	+	85.7%	low
Belcadi, 2022 [20]	CR	+	+	+	-	+	+	+	+	87.5%	low
Bonné, 2020 [21]	CR	+	+	+	+	+	+	+	+	100%	low
Buljan, 2018 [15]	CR	+	+	+	-	+	+	U	+	85.7%	low
Campos-Muñoz, 2007 [8]	CR	+	+	+	-	+	U	U	+	83.3%	low
Costa, 2014 [22]	CR	+	+	+	+	+	+	U	+	100%	low
De Giorgi, 2003 [24]	CR	+	+	+	+	+	+	+	+	100%	low
Dong, 2016 [16]	CR	+	+	+	-	U	+	+	+	85.7%	low
Drakensjö, 2012 [9]	CR	+	+	+	-	+	+	+	+	87.5%	low
Gallouj, 2014 [36]	CR	+	+	+	-	+	+	+	+	87.5%	low
Garrido Colmenero, 2015 [17]	CR	+	+	+	-	+	+	+	+	87.5%	low
Ge, 2016 [18]	CR	+	+	+	-	+	+	+	+	87.5%	low
Gracia-Darder, 2022 [7]	CR	-	-	+	-	-	-	-	-	12.5%	high
Ha, 2021 [6]	CSS	+	+	+	+	U	U	+	-	83.3%	low
Hamich, 2022 [38]	CR	+	+	+	-	+	+	U	+	85.7%	low
Inskip, 2016 [14]	CR	+	+	+	+	+	+	U	+	100%	low
Jaime, 2013 [25]	CR	+	+	+	+	+	+	+	+	100%	low
Jassi, 2020 [32]	CR	+	+	+	+	-	+	U	+	85.7%	low
Jouini, 2022 [37]	CR	+	+	+	-	+	+	U	+	85.7%	low
Kołcz, 2023 [35]	CR	+	+	+	-	+	+	U	+	85.7%	low
Kurosaki, 2023 [13]	CR	+	+	+	+	+	+	+	+	100%	low
Levakov, 2020 [26]	CR	+	+	+	-	+	+	+	+	87.5%	low
Martos-Cabrera, 2023 [34]	CR	+	+	+	-	+	+	U	+	85.7%	low
Mun, 2013 [19]	CR	+	+	+	-	+	+	+	+	87.5%	low
Nam, 2020 [39]	CR	+	+	+	-	+	+	+	+	87.5%	low
Ramirez, 2011 [11]	CR	+	+	+	-	+	+	+	+	87.5%	low

Resuello, 2022 [33]	CR	+	+	+	-	+	+	+	+	87.5%	low
Sandoval, 2021 [27]	CR	+	+	+	+	+	+	+	+	100%	low
Siebel, 2014 [30]	CR	+	+	+	-	+	+	U	+	85.7%	low
Takada, 2021 [12]	CR	+	+	+	-	+	+	+	+	87.5%	low
Vega-Castillo, 2022 [28]	CR	+	+	+	+	+	+	+	+	100%	low
Wobser, 2009 [29]	CR	+	+	+	-	+	+	U	+	85.7%	low

JBI – Joanna Briggs Institute; Q – question; CR – case report tool; CSS – analytical cross sectional study tool; U – unclear
