# Supplementary material for: Dermoscopy of Umbilical Lesions—A Systematic Review
Source: J Clin Med. 2024 Mar 20;13(6):1790. doi: 10.3390/jcm13061790 (PMC10970748; doi:10.3390/jcm13061790)
Supplement: Supplementary file 1 [file jcm-13-01790-s001.zip › Supplementary_Table_S3.pdf]

Supplementary Table 3. Overview of the characteristics of included studies.

Diagnosis	First author, journal, year	Dermoscopic features	Corresponding IDS terminology	Histological confirmation	Histological background of the findings	Dermoscope (polarized/non-polarized) – magnification (immersion)	Number of cases	Study design/ Oxford level of evidence	Comments
**Melanocytic lesions**									
Melanoma	Campos-Muñoz et al., Clin Exp Dermatol., 2007	Homogeneous blue-grey background with a superimposed atypical pigment network at the centre.<br><br>Brownish pigmentation with atypical pigment network.<br><br>Dark brown globules at the periphery.	Structureless zone, blue, gray<br><br>Lines reticular and thick or reticular lines that vary in color<br>Clods, brown, small	Yes	Breslow depth: 1.06 mm<br>Clark level: III<br>Many dermal melanophages: blue-grey background.<br>Enlargements of the rete ridges: atypical pigment network.	NR (NR) – NR (NR)	1	Case report/V	
Congenital melanocytic naevus	Drakensjö et al., Acta Derm Venereol., 2012	Irregular pigment distribution with confluence of black and brown dots.	Dots, brown, black	No	NR	NR (NR) – NR (NR)	1	Case report/V	Spontaneous regression of the lesion – residual brown macula and a greyish shadow after 3 years of follow-up.
Intradermal nevus	Bandeira et al., An Bras Dermatol., 2018	Comma-shaped and irregular vessels on a light pink background.<br><br>Surrounding serous and hematic crust.	Curved vessels<br>Structureless zone, pink<br><br>No corresponding IDS terminology	Yes	Epidermal atrophy; nevus cells without atypia in the dermis; preserved deep maturation, interspersed with congested blood vessels.	NR (NR) – NR (NR)	1	Case report/V	Ulcerated intradermal nevus. Dermoscopy of cerebriform area. Cerebriform area surrounded by serous-hematic crust.
**Non-melanocytic malignant lesions**									
Basal cell carcinoma	Ramirez et al., Dermatol. Online J., 2011	Semi-translucent appearance.<br><br>Small superficial ulceration.<br><br>Polymorphous, in focus vessels: hairpin vessels, prominent arborizing vessels.<br><br>Fine elongated telangiectasias.	No corresponding IDS terminology<br>No corresponding IDS terminology<br>Polymorphous vessels: looped, branched<br>No corresponding	Yes	NR	NR (NR) – NR (NR)	1	Case report/V	Found during complete body mole mapping.

				IDS terminology						
	Takada et al., An Bras Dermatol., 2021		Large blue-gray ovoid nests.	Clods, blue, large, clustered	Yes	Atypical cells forming nests extending from the epidermis with a palisading pattern and a small number of melanin deposits. Artifactual clefting around the nest.	NR (NR) – NR (NR)	1	Case report/V	
	Kurosaki et al., Medicine, 2023	Case 1.	Blue-gray globules.<br>Large blue-gray ovoid nests.	Clods, blue, small<br>Clods, blue, large	Yes	Atypical cells contiguous with the epidermis forming a nest with a fenestrated arrangement at the margins.	NR (NR) – NR (NR)	2	Case report/V	
		Case 2.	Large blue-gray ovoid nests.<br>Arborizing vessels.	Clods, blue, large<br>Branched vessels		No specific structures reported.				
Fibroepithelioma of Pinkus	Inskip et al., Dermatol Pract Concept., 2016	Lesion 1. (nonpigmented)	Multiple small erosions<br>Fine polymorphic peripheral vessels on a pink/white background.	No corresponding IDS terminology<br>Polymorphous vessels<br>Structureless zone, pink, white	Yes	Interconnecting and ramifying cords of basaloid epithelium set in a loose stroma; the infiltrative and arborizing pattern; macrophages within the intervening stroma (pigmented lesion).	Medicam 800 Fotofinder (non–polarized) – 20x (NR)	1	Case report/V	Apparent two tumors. Two different dermoscopic patterns of one tumor.
		Lesion 2. (pigmented)	Small ovoid gray structures.<br>Fine brown dots.<br>Arborizing vessels.<br>Single erosion.	Clods, gray, small, clustered<br>Dots, brown<br>Branched vessels<br>No corresponding IDS terminology						
Sister Mary Joseph nodule	Buljan et al., Australas J Dermatol., 2018		Diffuse glomerular and dotted vessels.<br>Milky-red structureless areas.<br>White lines (as a result of the papillomatous surface of the lesion).	Coiled vessels, dots<br>Structureless zone, pink<br>Lines, white	Yes	NR	NR (NR) – NR (NR)	1	Case report/V	Gastric adenocarcinoma

	Dong et al., Australas J Dermatol., 2016	Aggregated frogspawn appearance with grey, yellow-grey and pink-grey coloration, and sometimes with a vessel in each 'egg' in the center; polymorphous vessels inside 'eggs' (irregular linear, arborizing, glomerular vessels).<br><br>Peripherally structureless pink area.	Polymorphous vessels (serpentine, branched, coiled)<br>Specific clue: 'frogspawn appearance'<br><br>Structureless zone, pink	Yes	Well-formed glandular structures in the superficial dermis, with balloon-shaped structures in the dermal papillae; single cancer cells or clusters of cancer cells in the deeper adjacent dermis.	NR (NR) – 10x (NR)	1	Case report/V	Gastric adenocarcinoma
	Garrido Colmenero et al., Actas Dermosifiliogr., 2015	White shiny structures.<br><br>Milky-red areas.<br><br>Atypical polymorphous vessels (serpentine, curved vessels).	Shiny white structures<br>Structureless zone, pink<br>Polymorphous vessels (serpentine, curved)	Yes	Subcutaneous infiltration by a metastasis from an adenocarcinoma.	NR (polarized) – NR (NR)	1	Case report/V	Colon cancer
	Ge et al., Dermatol Sin., 2016	Polymorphous vessels (serpentine, dotted, comma-shaped vessels).<br><br>White veil.	Polymorphous vessels (serpentine, dots, curved)<br>No corresponding IDS terminology	Yes	Diffuse infiltration of hyperchromatic and pleomorphic tumor cells in the dermis with a grenz zone.	NR (NR) – NR (NR)	1	Case report/V	Esophageal squamous cell carcinoma
	Gracia-Darder et al., J Cutan Med Surg., 2022	Milky-red structureless areas.<br><br>Yellow crust.<br><br>White lines.<br>Atypical polymorphic vascular pattern.	Structureless zone, pink<br>No corresponding IDS terminology<br>Lines, white<br>Polymorphous vessels	No	NR	NR (NR) – NR (NR)	1	Case report/V	
	Ha et al., Clin Med Insights Oncol., 2021	Polymorphous vessels.<br><br>White, milky-red structureless area.	Polymorphous vessels<br><br>Structureless zone, white, pink	Yes	NR	DermLite II Pro HR (NR) – 10x (NR)	1	Cross-sectional study/IV	1 out of 2 reported cases was included in the analysis as detailed frequency of dermoscopic features was not provided by the authors.
	Mun et al., J Am Acad Dermatol., 2013	Polymorphous vessels (linear serpentine, linear curved vessels).<br><br>White and milky-red structureless area.	Polymorphous vessels (serpentine, curved)<br><br>Structureless zone, white, pink	Yes	Metastatic adenocarcinoma. Increased vascularity around neoplastic cell nests in the papillary dermis: vascular pattern in dermoscopy.	NR (NR) – 10x (NR)	1	Case report/V	Pancreatic cancer

Mycosis fungoides	Belcadi et al., JAAD Case Rep., 2022	Comedo-like openings.<br><br>Black structureless area.	Clods, black<br><br>Structureless zone, black	Yes	Epidermotropic lymphoid infiltrate composed of medium-sized lymphocytes with cribriform notched nuclei and eosinophilic cytoplasm arranged in rows and theca. Fibrous dermis, with above-described cells arranged in a subepidermal band.	NR (NR) – NR (NR)	1	Case report/V	
**Non-melanocytic benign lesions**									
Dermatofibroma	Ha et al., Clin Med Insights Oncol., 2021	Pigment network.<br><br>White structureless area.	Lines, reticular<br><br>Structureless zone, white	Yes	NR	DermLite II Pro HR (NR) – 10x (NR)	1	Cross-sectional study/IV	1 out of 5 reported cases was included in the analysis as detailed frequency of dermoscopic features was not provided by the authors.
Endometriosis	Amaral Couto et al., Int J Clin Case Rep Rev., 2020	Polypoid projections in white color.<br><br>Grayish area around polyploid projections.<br><br>Central black dots.<br><br>Peripheral mild erythema.	Specific clue: polyploid projections in white color<br>Structureless zone, gray<br>Dots, black, central<br>No corresponding IDS terminology	Yes	Cystic shaped skin fragments, delineated by a single layer of cylindrical cells, presence of hemosiderin, inflammatory cells and fibrosis.	NR (polarized) – NR (NR)	1	Case report/V	
	Bonné et al., J Eur Acad Dermatol Venereol., 2020.	Three polypoid nodules; drainage hole on the top of each nodule; one drainage hole filled with amorphous red material; purplish-blue homogenous and poorly delimited area around the drainage holes.<br><br>Flesh-coloured, light to dark brown structureless areas.	Specific clues: polyploid nodules, drainage hole on the top of each nodule<br><br>Structureless zone, purple, blue, brown	Yes	Glandular structures in the superficial dermis, covered with cylindrical epithelium, with a lumen containing an eosinophilic substance and surrounded by a cytogenic stroma associated with a discrete	DermLite DL4 (NR) – 10x (immersion)	1	Case report/V	

					lymphocyte infiltration.				
	Buljan et al., Australas J Dermatol., 2018	Diffuse and homogeneously distributed dotted vessels.<br>Milky-red structureless area with a brownish hue.	Dots<br>Structureless zone, pink, brown	Yes	NR	NR (NR) – NR (NR)	1	Case report/V	
	Costa et al., Int J Dermatol., 2014	Follicular phase: Polypoid projections of erythematous violaceous color; active bleeding.<br>Areas with dark brown spots.	Specific clues: polyploid projections of erythematous violaceous color, active bleeding<br>No corresponding IDS terminology	Yes	Endometrial, glandular, and stromal elements in the reticular dermis.	Dermlite DL3 (polarized) – 10x (NR)	1	Case report/V	Lesion appeared two years after laparotomic tubal ligation in the umbilical scar.
		Luteal phase: Volume increase in the polypoid projections; increase in the number and size of the dark brown spots; areas with dark material.	No corresponding IDS terminology						
	De Giorgi et al., Clin Exp Dermatol., 2003	Homogeneous reddish pigmentation, regularly distributed, gradually fading toward the periphery.<br>Small red globular structures within typical pigmentation, but more defined and of a deeper hue, called 'red atolls'.	Structureless zone, red<br>Specific clue: 'red atolls'	Yes	Red atolls – multiple irregular glands containing erythrocytes embedded in a cellular stroma. Homogeneous reddish hue – myxoid, vascular stroma containing extravasated erythrocytes.<br>Multiple irregular glands embedded in a cellular stroma within the dermis. The glands were lined with a columnar epithelium with evidence of decapitation secretion, suggestive of the secretory phase of	Heine 10 (NR) – NR (immersion oil)	1	Case report/V	

					the menstrual cycle. Some of the glands were dilated and contained erythrocytes and cell debris. The endometrial glands were surrounded by a myxoid, vascular stroma containing extravasated erythrocytes and haemosiderin depositions.				
	Jaime et al., An Bras Dermatol., 2013	Homogeneous reddish localized pigmentation.<br><br>Amorphous brown area.<br><br>Regular pigmented skin network in the center.	Structureless zone, red, brown<br>No corresponding IDS terminology<br>Lines, reticular, central	Yes	Fibroblastic proliferation in the dermis and endometrial glandular tissue, of the decapitation secretion type. Deposit of hemosiderin in the dermis.	DermLite II Pro HR (polarized) – 10x (NR)	1	Case report/V	
	Levakov et al., Acta Dermatovenerol Croat., 2020	Small red globular structures ("red atolls") within regularly distributed homogeneous reddish pigmentation fading at the periphery.	Specific clue: 'red atolls'<br>Structureless zone, red	Yes	Endometriosis of cutaneous and subcutaneous adipose tissue.	NR (NR) – NR (NR)	1	Case report/V	
	Sandoval et al., Australas J Dermatol., 2021	Pink homogeneous lesion with a focal bluish blotch/clod.	Structureless zone, pink<br>Clods, blue	Yes	Connective tissue with a nodular area composed of normotypic endometrial glands surrounded by endometrial stroma with interstitial erythrocytes and hemosiderin-loaded macrophages.	NR (polarized) – NR (NR)	1	Case report/V	
	Vega-Castillo et al., Dermatol Pract Concept., 2022	Central white reticular pattern on a violet background.	Lines, white, central<br>Structureless zone, violet	Yes	NR	DermLite DL4 (polarized) – 10x (NR)	1	Case report/V	
	Wobser et al., Geburtshilfe und Frauenheilkunde, 2009	Red globules.<br><br>Telangiectasias.	Clods, red, small<br>No corresponding IDS terminology	Yes	Glandular tissue with cubic epithelium within a mucinous stroma.	NR (NR) – NR (NR)	1	Case report/V	

		Diffuse homogenous milky-red area.	Structureless zone, pink						
Epidermal cyst	Ha et al., Clin Med Insights Oncol., 2021	The ‘pore’ sign.	Specific clue: the ‘pore’ sign	Yes	NR	DermLite II Pro HR (NR) – 10x (NR)	1	Cross-sectional study/IV	
Epidermal nevus	Ha et al., Clin Med Insights Oncol., 2021	Diffuse pigmentation.	No corresponding IDS terminology	Yes	NR	DermLite II Pro HR (NR) – 10x (NR)	1	Cross-sectional study/IV	
	Siebel et al., Surg Cosmet Dermatol., 2014	Yellow-orange color areas in the center.<br>Peripheral verrucous lesions (on the wall of the umbilical scar).<br>Fine regular pigmented network in the margins of the lesion.<br>Debris.	Structureless zones, yellow, orange<br>No corresponding IDS terminology<br>Lines reticular<br>No corresponding IDS terminology	Yes	Laminar hyperkeratosis; acanthosis; papillomatosis. Dermal fibrosis: umbilical scar.	NR (NR) – NR (NR)	1	Case report/V	Verrucous epidermal nevus
Granuloma	Ancer-Arellano et al., Pediatr Dermatol., 2019	Linear irregular vessels.<br>Arborizing vessels.<br>Structureless areas over a milky-red background.	Linear vessels<br>Branched vessels<br>Structureless zone, pink	No	NR	DermLite DL3N (polarized) – 10x (NR)	1	Case report/V	
	Jassi et al., Indian Dermatol Online J., 2020	Fine linear vessels.<br>Dotted vessels.<br>Tortuous vessels.<br>Milky-white background.	Linear vessels<br>Dots<br>No corresponding IDS terminology<br>Structureless zone, pink	No	NR	DermLite 3N (polarized) – 10x (NR)	1	Case report/V	
Intravascular Papillary Endothelial Hyperplasia	Resuello et al., Diagn Pathol., 2022 (under review)	Patchy pseudo-reticular network with thick brown lines surrounding a structureless area.<br>Milky-red structureless area.<br>White veil.<br>Multiple shiny white lines and strands.	Lines, reticular and thick<br>Structureless zone, pink<br>No corresponding IDS terminology<br>Shiny white lines	Yes	Dilated vascular spaces lined by pleomorphic cells exhibiting mild atypia.	NR (NR) – NR (NR)	1	Case report/V	
Lichen planus	Martos-Cabrera et al., An Bras Dermatol., 2023	White streaks on a violaceus background.	Lines, white, perpendicular<br>Structureless zone, violet	Yes	Hyperkeratosis and cytoid bodies with a bandlike inflammatory cell infiltrate composed of lympho- cytes, histiocytes, and occasional	NR (NR) – NR (NR)	1	Case report/V	

					eosinophils in the papillary dermis.				
	Kołcz et al., Forum Dermatologicum, 2023	Irregular pinkish structureless areas intermingled with whitish lines.<br>Grey dots.<br>Grey-black structureless area.<br>Peripheral white lines resembling Wickham striae.	Structureless zone, pink<br>Lines, white<br>Dots, grey<br>Structureless zone, grey-black<br>Lines, white	Yes	Hypertrophic stratum corneum and granular layer, the proliferation of the squamous layer with the characteristic “saw tooth” appearance, hydrotic degeneration, presence of cytoid bodies, melanophages and melanin incontinence.<br>Band-like lymphocytic-histiocytic inflammatory infiltrate in the superficial dermis.	NR (NR) – NR (NR)	1	Case report/V	Lichen planus pigmentosus inversus
Omphalolith	Gallouj et al., Dermatol Online J., 2014	Dry crusted appearance.<br>Black-brown structureless areas.	No corresponding IDS terminology<br>Structureless zone, black, brown	Yes	Laminated keratin; amorphous material resembling sebum; numerous terminal hairs; scattered collections of bacteria.<br>Melanin and oxidized lipids: black color.	NR (NR) – NR (NR)	1	Case report/V	
	Jouini et al., Clin Case Rep., 2022	Dry crusted pigmented lamellar keratotic material.	Structureless zone, brown, yellow	No	NR	NR (NR) – NR (NR)	1	Case report/V	
Seborrheic Keratosis	Hamich et al., Clin Med Img Lib., 2022	Exophytic keratotic projection pattern.<br>Dotted vessels.<br>Brown and yellowish structureless areas.<br>Aggregated blue-gray globular-like structures.	Specific clue: ‘exophytic keratotic projection pattern’<br>Dots<br>Structureless zone, brown, yellow<br>Clods, blue, grey, clustered	Yes	Epidermal proliferation with a hyperplastic epidermis surrounded by a layer of orthokeratotic and papillomatous keratin with a proliferation made mainly of epidermal basal cells.	NR (NR) – NR (NR)	1	Case report/V	
Syringoma	Nam et al., Ann Dermatol., 2020	Multiple pink to whitish areas surrounded by finely pigmented network.	Structureless zone, pink, white<br>Lines, reticular	Yes	Multiple ductal and small cystic structures embedded within	NR (NR) – NR (NR)	1	Case report/V	

					a fibrous connective tissue stroma. The ducts were lined by 1 to 2 rows of epithelial cells. Some ducts showed a "tadpole"-like appearance.				

NR – not reported
